# MMP9: A Tough Target for Targeted Therapy for Cancer

**DOI:** 10.3390/cancers14071847

**Published:** 2022-04-06

**Authors:** Katarzyna Augoff, Anita Hryniewicz-Jankowska, Renata Tabola, Kamilla Stach

**Affiliations:** 1 Department of Surgical Education, Wroclaw Medical University, 50-367 Wroclaw, Poland; 2 Department of Chemistry and Immunochemistry, Wroclaw Medical University, 50-367 Wroclaw, Poland; kamilla.stach@umw.edu.pl; 3 Deprtment of Cytobiochemistry, Biotechnology Faculty, University of Wroclaw, 50-383 Wroclaw, Poland; anita.hryniewicz-jankowska@uwr.edu.pl; 4 Department of Thoracic Surgery, Wroclaw Medical University, 50-367 Wroclaw, Poland; renata.tabola@umw.edu.pl

**Keywords:** matrix metalloproteinase 9, extracellular matrix, epithelial–mesenchymal transmission, cancer-related inflammation, targeted therapy

## Abstract

**Simple Summary:**

MMP9, due to its proteolytic activity, plays a key role in tumorigenesis by regulating migration, epithelial-to-mesenchymal transition and survival of cancer cells, induction of immune response, angiogenesis and formation of tumor microenvironment. It seems to be an attractive target for anticancer therapies. However, on the one hand very complicated mechanism of regulation of expression, synthesis and activation of MMP9, which determines specific, often contradictory role of this enzyme, and on the other hand high homology with other members of MMP family, makes development of effective and safe MMP9 inhibitors as anticancer drugs extremely difficult. Recently, blocking antibodies that selectively inactivate MMP9 have raised hopes and are currently in clinical trials. However, there is still a need to deepen our understanding of the mechanisms by which MMP9 becomes an important part of the tumorigenesis process. The goal is to develop a new generation of therapies that target MMP9 for the treatment of cancer.

**Abstract:**

Having the capability to proteolyze diverse structural and signaling proteins, matrix metalloproteinase 9 (MMP9), one of the best-studied secretory endopeptidases, has been identified as a crucial mediator of processes closely associated with tumorigenesis, such as the extracellular matrix reorganization, epithelial to mesenchymal transition, cell migration, new blood vessel formation, and immune response. In this review, we present the current state of knowledge on MMP9 and its role in cancer growth in the context of cell adhesion/migration, cancer-related inflammation, and tumor microenvironment formation. We also summarize recent achievements in the development of selective MMP9 inhibitors and the limitations of using them as anticancer drugs.

## 1. Introduction

Malignant neoplasms are considered a major and still-growing global health problem. According to GLOBOCAN, around 18.3 million new cases and almost 10 million cancer-related deaths occurred worldwide in 2020 [1]. The development of neoplastic tumors is a complex, multifaceted process in which the role of mutual interactions between cancer cells and the local benign environment seems to be important. There is ample evidence that extracellular proteinases, such as the matrix metalloproteinases (MMPs), serve as key modulators of cell–cell and cell–extracellular matrix communication that protect tissue homeostasis. Thanks to the ability to liberate bioactive peptides and growth factors, and to degrade proteins on both the cell surface and extracellular proteins, they can have a decisive influence on the fate and behavior of cells, as well as on the organization of the stroma. Remarkable upregulation of gene expression and the activity of MMPs, especially MMP9, has been observed in various malignancies [2,3,4]. As it was found that elevated MMP9 levels are positively correlated with a poor prognosis in cancer patients, MMP9 has been suggested as a potential marker candidate for such cancers as breast, colorectal, ovarian, and non-small cell lung cancer [3,4,5,6]. In this context, the inhibition of MMP9 activity meets the criteria of an attractive anticancer therapy strategy. Broad-spectrum inhibitors of MMP9 have been developed, mainly small molecules, and several have been evaluated in clinical trials. However, all of them failed mainly due to dose-limiting toxicity, lack of selectivity of the inhibitor, or insufficient efficacy, making the treatment difficult to tolerate by patients. While the recent development of therapeutic antibodies to MMP9 offers a strategy to overcome some of these problems, inhibition efficacy is still lacking. Hence, there is a need to continue the search for selective, safe, and more effective MMP9 inhibitors, as well as to broaden the knowledge about the mechanisms regulating the expression and activation of MMP9 in order to use them as an important tool in anticancer therapy.

Here, we review the current understanding of MMP9, its regulation and biochemical properties, and its role in cancer progression. We also summarize recent advances in the development of selective MMP9 inhibitors and the limitations of their use as anticancer drugs.

## 2. Matrix Metalloproteinase 9: A Secreted Member of the Zinc Metalloendopeptidase Family

### 2.1. Biochemical Characteristics of MMP9

**Figure 1:**
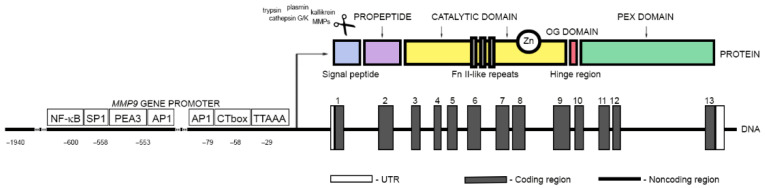
A schematic representation of the MMP9 gene and protein organization. The exons are indicated by the numbered grey rectangles. The 5′-untranslated and 3′-untranslated regions (UTR) are indicated by white boxes. The 5′-flanking region includes relative positions of key transcription factor binding sites. The domain structure of the MMP9 protein starts from a signal peptide (lilac square), followed by a propeptide (blue rectangle). An O-glycosylated (OG) domain (red rectangle) is located between a catalytic domain and a hemopexin (PEX) domain (green rectangle); it comprises a zinc-binding active-site motif (Zn) and tree fibronectin-like (Fn II-like) repeats.

Matrix metallopeptidase 9, also known as gelatinase B or 92 kDa type IV collagenase (EC 3.4.24.35), is one of the two gelatinases that belong to the matrix metalloproteinase (MMP) family of zinc metalloendopeptidases (Supplementary Table S1). It is a multidomain protein with molecular weight of 92 kDa that catalyzes the degradation of large extracellular matrix (ECM) components, such as elastin and collagens, as well as certain cell surface proteins. It is also involved in the release and activation of extracellular signaling molecules. MMP9 is synthesized and secreted as a zymogen with an amino-terminal propeptide, PRCGXPD, in which the cysteine sulfhydryl group chelates the zinc in the active site and keeps the MMP9 in its latent form (Figure 1). The latent form of MMP-9 was found to exist in two major forms, as a monomer and a disulfide-bonded homodimer [7].

Removal of the prodomain is necessary for MMP9 activation and can be mediated by several proteases, such as trypsin, plasmin, cathepsin G or K, kallikrein, and other metalloproteases/metalloproteinases as well as mercurial compounds, chaotropic agents, oxidants, disulfide compounds, and alkylating agents [8,9,10,11,12]. The 160-residue zinc- and calcium-dependent catalytic domain of MMP9 (Phe107–Gly444) comprises a zinc-binding active-site motif represented by a consensus sequence, HE(FG)H(AL)G(LD)H; two Zn^2+^ and five Ca^2+^ ions; and three fibronectin type II (FnII)-like repeats that enhance enzyme–substrate interactions. MMP9 also contains a unique 54-amino acid insert, known as the O-glycosylated (OG) domain, which links the catalytic domain with a regulatory subunit, the 209-amino acid residue C-terminal hemopexin (PEX) domain. [7,13]. This linker is rich in prolines and has low homology to the α2 chain of type V collagen. It is also heavily O-glycosylated. Its role is not clear, although it was found to be required for maintaining the proper orientation of the hemopexin domain for the tissue inhibitor of metalloproteinases-1 (TIMP-1) binding and internalization of MMP9 by both low-density lipoprotein receptor-related protein (LRP) and megalin [14]. The hemopexin domain of MMP9 is responsible for the improvement of the selectivity and specificity of substrate binding. It is also involved in the activation and inhibition of MMP9 and homodimerization [13,15]. MMP9 was shown to have two N-glycosylation sites on asparagine residues at position 38 in the propeptide domain (Asn38) and in the catalytic domain at position 120 (Asn120). The role of N-glycosylation in MMP9 is not fully understood. It appears not to be critical for MMP9 activation or its proteolytic activity, but it seems to be important for MMP9 protein structure stabilization and secretion [16,17,18]. Dimerization of MMP9 also appears to be independent of glycosylation [19].

### 2.2. Transcriptional and Post-Transcriptional Regulation of MMP9 Gene Expression

The expression of MMP9 is regulated at both the transcriptional and post-transcriptional level by various *trans*-activators or noncoding RNAs, respectively, as well as by epigenetic mechanisms, such as DNA methylation and histone modifications. All these mechanisms can contribute to both the activation and repression of MMP9 gene expression [20,21]. The activation of a transcription is regulated by a complex process that closely depends on the ordered recruitment of transcription factors and chromatin remodeling factors to the promoter of a target gene. The promoter region of the MMP9 gene comprises several consensus binding motifs for regulatory transcription factors (TFs), including TATA motif-like sequence (TTAAA), GT-box (CACCC), activator protein-1 (AP-1) motif, a nuclear factor-κB (NF-κB)-like sequence, and multiple polyomavirus enhancer-binding activator-3 (PEA3) elements or GC-box (CCGCCC), the binding site for specificity protein 1 (Sp1) [22,23,24]. Regulatory proteins generally function cooperatively in specific combinations. For instance, the proximal AP-1 has been found to activate MMP9 gene expression in synergism with NF-κB and Sp1 [25,26]. Additionally, multiple different coactivators were found to be associated with the MMP9 promoter, including the cAMP-response-element binding protein (CREB)-binding protein (CBP) and p300 (CBP/p300), p300/CBP-associated factor (PCAF), coactivator-associated arginine methyltransferase 1 (CARM1), and a p160 steroid receptor coactivator (SRC) member, GRIP1. These cofactors are able to induce MMP9 promoter activity in independent, additive, and synergistic manners [27]. PCAF and CARM1, through their participation in histone modification and chromatin remodeling, were shown to allow the binding of transcription factors to the MMP9 promoter and provide docking sites for other transcriptional coactivators [27,28]. On the other hand, alterations in the availability of coactivators, such as in the case of the sequestration of PCAF under interferon-κ-induced STAT-1α activation, were found to suppress MMP9 gene transcription [29]. Recently, also the CDKN1A/p21 protein was identified as a potential transcription regulator of the tumor necrosis factor α-induced MMP9 gene expression [30]. Previously, Snowden et al. reported a close relationship between CDKN1A/p21 expression and the activation of CBP/p300-dependent transcription [31].

### 2.3. Extracellular Signal-Induced Upregulation of MMP9

**Figure 2:**
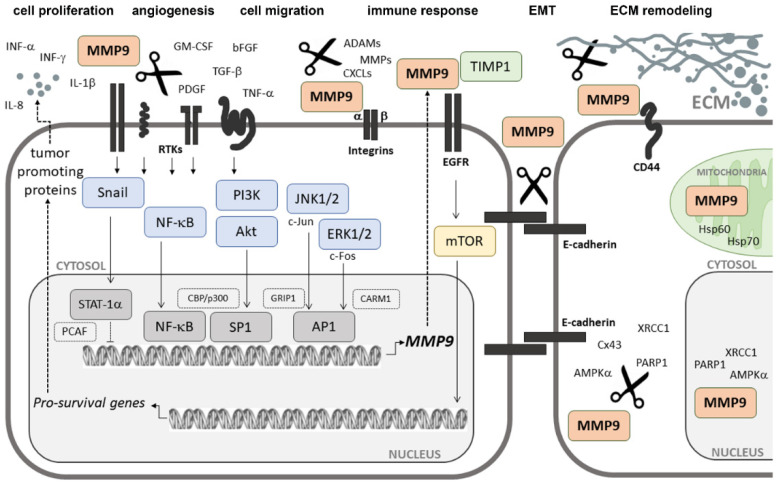
Schematic representation of the signaling pathways involved in MMP9 expression. The various extracellular signals, including proinflammatory cytokines (TNF-α, IL-8, and IL-1β) and growth factors (TGF-β, PDGF, and bFGF), can bind to their receptors and activate downstream signaling cascades involved in the activation of transcription factors (NF-κB, SP1, and AP1), which can than bind to specific sequences of the MMP9 gene promoter to trigger transcription. Transcription factors activate transcription in cooperation with additional transcriptional cofactors (CBP/p300, GRIP1, and CARM1). A secreted MMP9 protein degrades extracellular matrix proteins (collagens and elastin), cytokines (IL-1β, IL-8, and TNF-α), chemokines (CXCLs), and other matrix metalloproteases (MMPs). MMP9 by interactions with cellular receptors (CD44, E-cadherin, and α/β integrins) is able to proteolyze cell surface proteins. MMP9 activity in the extracellular matrix (ECM) can be regulated by its natural inhibitor TIMP-1. INFγ was found to inhibit the expression of MMP9 by STAT-1α-dependent sequestration of a PCAF cofactor. In this way, MMP9 regulates tissue remodeling, cell–cell and cell–ECM interactions, and activation of extracellular signal molecules, which promote cell migration, proliferation, angiogenesis, EMT, and ECM remodeling.

MMP9 is produced mainly by monocytes and inflammatory macrophages, as well as by neutrophilic granulocytes and most cancer cells. However, only in neutrophils MMP9 is stored in specific tertiary granules from where it can be rapidly released. In other cell types, MMP9 is synthesized de novo during stimulation induced with cytokines [32,33]. It is known that, aside from interferon-α(IFα), other different inflammatory cytokines and growth factors, including interferon-γ(IFNγ), tumor necrosis factor α (TNF-α), interleukin 1β (IL-1β) or granulocyte macrophage colony-stimulating factor (GM-CSF), transforming growth factor β (TGF-β), platelet-derived growth factor (PDGF), or basic fibroblast growth factor (bFGF), can regulate MMP9 secretion and activation [24,26,34,35,36,37,38,39]. Their effects on MMP9 are often dependent upon whether they act individually or in combination. For instance, IFNγ is known to inhibit TNF-α-induced upregulation of MMP9 in a range of human cell types, but in the presence of GM-CSF or with interleukin 1β (IL-1β) synergizes to increase MMP9 secretion [40,41]. Similarly, IL-1β was shown to interact synergistically with either PDGF or bFGF [19]. Depending on the specific stimuli and their cell surface receptors, diverse independent signaling cascades can be triggered and lead to the expression of MMP9 [42,43]. Protein kinases (PKs) belonging to the mitogen-activated protein kinase (MAPK) superfamily are indicated as major players in this process. Some of these proteins, including extracellular signal-regulated kinases (ERKs), ERK1 and ERK2, or c-Jun N-terminal kinases (JNKs), JNK1 and JNK2, are known to induce the expression of genes encoding c-fos (p62c-fos) and c-jun (p39c-jun) (Figure 2). The protein products of these genes are members of the AP-1 transcription factors involved in the transcriptional regulation of MMP9 [44]. The use of compounds selectively targeting the MEK/ERK pathway, such as U0126 or PD98059, was shown to significantly reduce MMP9 expression and activity [37,45]. It was also demonstrated that the use of a dominant negative mutant of ERK1, in which the conserved residue Lys71 was replaced by Arg, resulted in the reduction of MMP9 levels in glioblastoma cells [46]. However, among all signaling pathways, the nuclear factor kappa-light-chain-enhancer of activated B cell (NF-κB) activity seems to play a prime role for MMP9 expression and secretion [39,47]. A consensus sequence for binding NF-κB was identified exclusively for MMP9 and localized at ~600 nucleotides upstream of the transcriptional start site (TSS) within the human promoter region of the MMP9 gene [25,39,48]. The mutations introduced into the NF-κB binding motif were shown to markedly reduce, if not entirely block, the basal responsiveness of the promoter to MMP9 inducers, such as phorbol ester and TNF-α or even to TGF-β [39,49].

### 2.4. MMP9 Synthesis and Activation

MMP9 is synthesized as a preproenzyme containing a 19-amino-acid-long signal peptide, and before it is secreted, it enters the endoplasmic reticulum (ER), in which it undergoes maturation through a stepwise glycosylation with an 85 kDa underglycosylated precursor form and an 89 kDa intermediate glycosylated form. The 92 kDa fully glycolyzed form is produced in the Golgi apparatus and transported to the plasma membrane in a Rab3D-dependent manner by small Golgi-derived cytoplasmic vesicles or gelatinase granules [50]. With a few exceptions, including neutrophils, most cells secrete MMP9 in a noncovalent complex with TIMP-1, an important regulator of MMP9 activation [51,52]. It was found that both activated and latent forms of the enzyme were able to bind to TIMP-1. The interactions between the latent form of MMP9 and TIMP-1 are mediated through the C-terminal hemopexin-like domain of MMP9 and the C-terminus of TIMP-1. The N-terminal domain of TIMP-1 is known to interact with and inhibit the active form of MMP9 [52,53]. Human neutrophil MMP9, which is not secreted in a complex with TIMP-1, was found to form a 125 kDa disulfide-linked heterodimer with neutrophil gelatinase-associated lipocalin (NGAL), also known also as lipocalin 2 (Lcn2) or p25 [54]. The disulfide bridge in this complex is formed between free Cys87 of the NGAL protein and one of the cysteine residues residing in the hemopexin domain of MMP9. Though, as was recently found, only a part of neutrophil MMP9 forms complexes with NGAL and these two proteins exist primarily independently of each other, NGAL was shown to stabilize MMP9 and enhance the activation of its latent form [55]. NGAL was also found to modulate MMP9 activity by protecting it from proteolytic degradation [56].

During activation, the 92 kDa latent form of MMP9 undergoes two successive proteolytic cleavages. The first one occurs at the Glu59-Met60 bond located in the turn that connects helix 1 and helix 2 of the propeptide, generating an 86 kDa intermediary form. The other cleavage occurs eight amino acid residues downstream from the zinc coordinating Cys99 at the Arg106–Phe107 position, yielding an 82 kDa active species [9,57,58]. These cleavages are required for disrupting the zinc–cysteine complex present within the proenzyme domain to expose the catalytic site of the enzyme. Recently, it was reported that N-glycosylation had an important impact on the order of these proteolytic steps. Using molecular dynamics (MD) simulations, Kumar et al. demonstrated that the presence of a glycan chain at Asn38 within the MMP9 prodomain induces conformational changes in the region of the first cleavage in such a way that they prevent the other cleavage from being processed first [18]. Further processing of the 82 kDa form of MMP9, as a result of the prolongated exposure to 4-aminophenylmercuric acetate (APMA) or matrix metalloproteinase 3 (MMP3), can lead to a final, still-active 67 kDa product, lacking the C-terminal fragment, or an inactive product of 50 kDa devoid of the catalytic domain [59]. The removal of the peptide fragment that contains a conserved Cys residue from the 86 kDa intermediary form is usually related to proteolytic processes. However, it was shown that mere binding of the proenzyme to a ligand or to a substrate may disrupt the cross-link between Cys and the active center of the enzyme, causing its activation [60]. 

### 2.5. Substrate Specificity of MMP9

MMP9 is known to have a wide range of physiologic extracellular matrix and nonmatrix substrates including different types of collagens such as types I, III, IV, V, and XI or cytokines, chemokines, growth factors, and their receptors that are crucial in mediating physiological and pathological processes. MMP9 is best known for its highly efficient proteolytic activity against gelatin, a heat-denatured form of collagen [61]. Bigg et al., using recombinant human MMP9 expressed in insect cells, showed that MMP9, like other collagenases, is able to cleave solubilized, native types of collagen monomers, generating the typical 3/4 (TC^A^) and 1/4 (TC^B^) length collagen fragments [62]. These findings were confirmed by Rosenblum et al., who described the mechanism of collagenolytic activity of MMP9 in detail [63]. 

The use of a phage display method has led to the identification of three groups of specific substrates for MMP9 that are defined by different motifs and developed different ways to bind with MMP9. Peptides containing a Pro at P_3_ and a large hydrophobic residue at P_1′_ (Pro-X-X-Hy, where X is any residue) were found to be cleaved most efficiently by MMP9 and established group I of substrates comprising all collagens known to be cleaved by MMP9. It was shown that MMP9 also had unique preferences for Ser/Thr at P_2′_ within these substrates (Pro-X-X-Hy-(Ser/Thr)). The other group of substrates for MMP9 has been defined by a Gly-Leu-(Lys/Arg) consensus motif at positions P_1_ to P_2_. Members of the third group of substrates were characterized by the presence of Arg at P_2_ and often at P_1_ positions (Pro-Arg-Arg-Hy) [64].

The proteolytic activity of MMP9 directed against major protein components of the extracellular matrix (ECM), including specialized structures, such as basement membranes (BMs), makes MMP9 a key regulator of extracellular space organization. Tightly controlled changes in the extracellular network of macromolecules are known to contribute to the normal tissue development, inflammation, and wound healing but also fibrosis or cancer growth and invasion [65,66]. Protease-regulated modifications of the ECM composition and structure allow cell migration and may also result in the release of biologically active factors or signaling molecules. In vivo studies using Mmp9−/− mice have demonstrated that MMP9 deficiency is associated with various abnormalities in processes that are relevant for cancer development, such as inflammation, wound healing, and vascular wall remodeling [67,68,69,70,71]. Shchors et al. observed that MMP9 gene deletion significantly impaired tumor growth and angiogenesis in two independent models of pancreatic neuroendocrine carcinogenesis. Ultimately, however, MMP9-deficient tumors of both models exhibited markedly more invasive phenotypes compared with their wild-type counterparts [72].

## 3. MMP9 and Cancer

### 3.1. MMP9 in Cancer Cell Adhesion and Migration

The spatial organization of the epithelium is precisely regulated by signals of cell–cell and cell–ECM adhesions. It has been reported that E-cadherin, a cell–cell adhesion molecule that is critical for maintaining the epithelial structure and tissue homeostasis, is a direct target of several MMPs, including MMP9 [73,74,75]. MMP9-related cutting of the E-cadherin extracellular domain results in the reduction of its surface levels and the loss of stable cell–cell junctions, apico-basal membrane polarity, and epithelial architecture, which allows cancer cells to escape from their primary sites. The strong correlation between decreased E-cadherin levels and increased MMP9 expression and clinicopathological parameters that has been observed in many human tumors, including ovarian, gastric, and esophageal cancers, may confirm that this complex interplay is of crucial importance for cancer development and progression [74,76,77].

The disruption of both adherens junctions (AJs) and cell polarity is a key hallmark of a process known as epithelial-to-mesenchymal transition (EMT) in which epithelial cells assume a mesenchymal phenotype characterized by increased migratory and invasive potential [78]. Decreased levels of E-cadherin have been shown to be frequently correlated with high histopathologic grade and poor overall survival in a number of human carcinomas [79]. Using epithelial ovarian cancer (EOC) cells, Symowicz et al. discovered that the processing of E-cadherin by MMP9 was functionally linked to collagen-mediated integrin signaling and was dependent on the nonreceptor tyrosine kinase Src activity [75]. Other studies reported the strong relationship between MMP9 and signals from αvβ6 and α5β1 integrins in cells stimulated with fibronectin [80,81,82]. These observations demonstrate the important role of MMP9 in the complex adhesive crosstalk that drives cell migration.

The cleavage of mature E-cadherin by MMP9 releases into the extracellular environment a soluble E-cadherin (sE-cad) fragment of about 80 kDa, which localizes to the exosome surface and, by retaining its binding ability, plays an important role as a paracrine/autocrine inducer of angiogenesis and cancer cell invasion [83,84]. The upregulation of sE-cad-positive exosomes in body fluids, such as blood and urea, was shown to be closely associated with inflammatory processes in cancers and correlated with histopathological grade, metastasis, recurrence, and survival in cancer patients [84,85,86]. sE-cad, by binding to receptor tyrosine kinases (RTKs), including members of the human epidermal growth factor receptor (EGFR or HER) and insulin-like growth factor-1 receptor (IGF-1R) families of receptors, and by activating the MAPK or phosphatidylinositol 3-kinase (PI3K)/Akt and mammalian target of rapamycin (mTOR) signaling pathways, may be involved in the regulation of the expression and synthesis of various proteins necessary in cell survival, proliferation, and metabolism [87]. On the other hand, it was found that the ligand-dependent activation of RTKs, such as HER-1, insulin-like growth factor type 1 receptor (IGF-1R), or vascular endothelial growth factor receptor 1 (VEGFR-1), which are frequently overexpressed in cancers, induced MMP9 expression through PI3K/Akt- and MAPK/ERK1/2-dependent signals, such as the signal transducer and activator of transcriptions 3 and 5 (STAT3 and STAT5), and blocking of RTK–ligand interactions, and prevented MMP9 upregulation and inhibited MMP9-mediated cancer cell invasion [38,74,88,89,90]. This indicates a complex mechanism of regulation of MMP9 synthesis and secretion and suggests that under certain conditions MMP9 may act in a feedback manner. It is also known that the PI3K/Akt and MAPK/ERK1/2 signaling cascades activated in response to HER-1 stimulation regulate the localization of the MMP9 protein to the cell surface in ovarian tumor cells [74,91]. Wolczyk et al. reported that the treatment of breast cancer cells with TNF-α, a proinflammatory cytokine, increased MMP9 concentrations in lipid rafts [37].

A study on Coco-2 cells revealed that MMP9 could disrupt tight junction (TJ) integrity and subsequently alter both cell polarity and epithelial barrier function [92]. A study by Chiu and Lai showed that MMP9 was able to degrade claudin-5, a key protein component of TJs, by the IκB-α/NF-κB/MMP9 signaling pathway [93]. Recent evidence indicated that the degradative activity of MMP9 against cell surface proteins is associated with the capability of MMP9 to bind transiently with some cell membrane receptor proteins [94].

Yu and Stamenkovic showed that MMP9 colocalized with the hyaluronan (HA) receptor CD44 on the mouse mammary carcinoma and melanoma cell surface. They found that interactions between CD44 and MMP9 promoted the proteolytic activity of MMP9 against type IV of collagen, which correlated with cell invasiveness and enhanced tumor growth in vivo, and disruption of the CD44–MMP9 complex reduced the migratory potential of cancer cells [95]. CD44–MMP9 complex formation, a process in which the PEX domain of MMP9 appeared to play a critical role, was found to initiate cross-talk between CD44 and HER-1 and, consequently, to trigger the activation of downstream effectors for cell migration [96]. Using confocal microscopy, it was revealed that MMP9, along with CD44, accumulated in extending lamellipodia and invadopodia of migrating cells [97,98].

The CD44 protein is a well-known cell surface receptor of the hyaluronan receptor family involved in cell–cell and cell–matrix adhesion, which functions as an initiator of cytoskeletal protein rearrangements and a regulator of lipid raft organization [97,99]. Hyaluronic acid (HA)-dependent clustering of CD44 was indicated as a docking site for MMP9 that concentrates its proteolytic activity locally at the cell membrane to direct cell migration. Additionally, CD44-anchored MMP9 is known to be able to cleave and activate latent TGF-β [94]. Activation of TGF-β1 by fibroblast-associated MMP9 was found to be crucial for fibroblast-to-myofibroblast differentiation, a key step in tumor stroma formation [100]. A study using three-dimensional (3D) cell culture technology showed that the inhibition of the cell surface-associated MMP9 activity reduced the TGF-β-induced cellular response in fibroblasts, resulting in diminished fibronectin (FN) gene expression and, consequently, impaired contraction of the surrounding matrix [101]. Silencing of the MMP9 gene expression was found to block TGF-β1-induced EMT and inhibited the migratory potential and invasiveness of esophagus squamous cell carcinoma (ESCC) cells as well as thyroid cancer (TC) cells [102,103,104]. On the other hand, once TGF-β1 is activated, it enhances the expression and synthesis of MMP9 by the Snail signaling pathway, thereby creating a positive autocrine regulatory loop modulation of tumor progression [105]. Another study colocalized MMP9 with Toll-like receptor 4 (TLR-4) at the cell membrane of lipopolysaccharide (LPS)-treated macrophages and dendritic cells. It was demonstrated that MMP9 in cooperation with neuraminidase-1 (Neu1) sialidase bound to TLR-4 to serve as a signaling platform that is essential for receptor activation upon ligand binding [106]. In a similar way, MMP9 was shown to regulate the activity of nerve growth factor (NGF)-induced transmembrane receptor tyrosine kinase A (TrkA), EGFR/HER, and insulin receptor (IR) signaling in cancer cells [107,108,109]. The TLR-4-dependent signaling pathway is best known for its key role in the inducing inflammation triggered by endogenous danger-associated molecular pattern (DAMP) molecules, released upon stress or death of a cell. When activated, they enhance the production of MMP9 via the TLR4/MYD88/NF-κB/AP-1 axis and increase the tumorigenic potential of cancer cells, promoting their immune evasion [110,111,112].

### 3.2. MMP9 in Cancer-Related Inflammation

It is now widely accepted that inflammatory processes are closely associated with tumorigenesis and play a significant role at every stage of cancer growth. For quite some time, inflammation has been listed as the seventh hallmark of cancer. Accumulating evidence indicates that immune response components, such as immune-related signals and immune cells, including both tumor-promoting regulatory T cells (T_reg_) or myeloid-derived suppressor cells (MDSCs), tumor-antagonizing effector T cells (including CD8+ cytotoxic T cells and effector CD4+ T cells), natural killer (NK) cells, dendritic cells (DCs), and tumor associated macrophages (TAMs), are an integral part of the microenvironment of most cancer tissues, even those that are not causally linked to inflammation [113]. MMP9 was found to be able to proteolyze extracellular signal proteins, mostly members of the CXC (C-X-C motif) chemokine family, regulating immune cell trafficking and, therefore, is considered as an important creator and coordinator of the tumor immune microenvironment (TIME). [114]. It was shown that the MMP9-dependent N-terminal selective cleavage of interleukin 8 (IL-8 or CXCL8), which generated the truncated IL-8(7-77) form, resulted in a significant increase in immune cell migration and activity. As was demonstrated, this 70-amino-acid fragment of IL-8 was characterized by significantly higher activity in binding to neutrophils and enhanced signaling through CXC chemokine receptor-1 (CXCR1) [115]. IL-8 is known to promote not only the trafficking of neutrophils or MDSCs into the tumor stroma but also to induce epithelial-to-mesenchymal transition in cancer cells [116]. Using CXCR1-blocking antibodies, Ginestier et al. demonstrated that the binding of IL-8 to CXCR1 on breast cancer stem cells (CSCs), by the activation of the FAK/Akt/FOXO3A pathway, regulated stem cell renewal and cell survival. IL-8-dependent activation of focal adhesion kinase (FAK) was also found to inhibit Fas-associated protein with death domain (FADD), a downstream effector of the Fas cell surface death receptor (FAS) cascade, leading to the disruption of proapoptotic FAS ligand (FAS-L)-FAS signaling [117]. On the other hand, it is known that IL-8 induces MMP9 expression and secretion in cancer cells, which positively correlates with their metastatic potential [118]. It is also known that IL-8 promotes neutrophil degranulation, which results in the release of TIMP1-free MMP9 and enhances angiogenetic activity [119]. Several other CXC chemokines, including CXCL1/GRO-α (growth-regulated alpha protein), CXCL4/PF-4 (platelet factor 4), CXCL5/ENA-78 (epithelial-derived neutrophil-activating peptide 78), CXCL7/CTAP-III (connective tissue-activating peptide III), CXCL9/MIG (monokine induced by interferon (INFγ)), CXCL10/IP-10 (INFγ-inducible protein-10), CXCL11/I-TAC (INF-inducible T cell α chemoattractant), and CXCL12/SDF-1 (stromal cell-derived factor 1), but not subfamily CC chemokines, have also been shown to be target substrates for MMP9 [115,120,121,122]. However, while MMP9-dependent cleavage of IL-8 and CXCL5/ENA-78 potentiated their biological activity, CXCL1/GRO-α, CXCL4/PF-4, CXCL7/CTAP-III, and CXCL12/SDF-1 were degraded by MMP9 to inactive fragments, losing their biological functions [115,120,123]. Therefore, MMP9 is able to reduce the chemotactic abilities of selected chemokines and hence inhibit the immune cell trafficking and tumor tissue infiltration.

CXCL5/ENA-78, commonly known as a strong neutrophil chemoattractant and a powerful mediator of inflammation, was shown to contribute to the promotion of cancer cell migration and invasion, associated with the activation of the EMT process by the ERK/GSK-3β/Snail pathway [124,125]. Van den Steen et al. reported that MMP9 proteolyzed the CXCL5/ENA-78 protein sequentially. They observed that complete degradation of CXCL5/ENA-78 into inactive fragments was preceded by the formation of highly active intermediates as a result of the removal of five to seven N-terminal residues [120]. Truncated forms of most CXC chemokines were characterized to be significantly more potent than their full-length variants; nevertheless, a proteolytic processing could release fragments with antagonistic properties [126]. N-terminal cutting of CXCL11/I-TAC by MMP9 was identified to generate the CXCL11/I-TAC(5-73) fragment, serving as a natural antagonist for CXC chemokine receptor 3 (CXCR3), which inhibits the activation of CXCR3-bearing lymphocytes, mainly effector T cells [122].

Aside from chemokines, some proinflammatory cytokines, such as TNF-α and IL-1β, were also found to be susceptible to cleavage by MMP9 [114]. Both TNF-α and IL-1β are key components of the cytokine network, which are known to regulate many different signaling pathways related to cell proliferation, cell survival, and death. TNF-α functions as a 26 kDa type II transmembrane protein (mTNF-α) on the surface of TNF-producing cells and as a soluble 17 kDa fragment (sTNF-α) that is cleaved from transmembrane TNF-α mainly by TNF-α-converting enzyme (TACE), also known as a disintegrin and metalloprotease 17 (ADAMS 17), and thus, it is able to manage the inflammatory response both as a ligand and as a cellular receptor [127]. A study using a recombinant TNF-α precursor revealed that a number of synthetic MMP inhibitors affected both the production and shedding of TNF-α, suggesting the involvement of many different MMPs in these processes. It was found that in the presence of MMP1, MMP3, and MMP7, as well as MMP9 and MMP2, the pro-form of TNF-α was cleaved, resulting in a 17 kDa product identical to the mature TNF-α form [128]. IL-1β is synthesized as a cell-associated 33 kDa inactive precursor that requires enzymatic cleavage to a biologically active form. MMP9 was shown to be capable of breaking down pro-IL-1β within minutes, releasing the active mature form of IL-1β [129].

In addition to its role in the activation of latent inflammatory molecules, MMP9 was identified as a potential inhibitor of immune response and immune cell trafficking by its ability to proteolyze receptors involved in the immune system. MMP9-dependent proteolytic cleavage of extracellular regions of tumor necrosis factor receptor 1 (TNFR1) and death receptor FAS/APO-1/TNFRSF6 (also known as CD95), known members of the TNFR superfamily (TNFRSF), has been recently shown to reduce chronic inflammation by the downregulation of cell-contact-related phagocytosis-induced cell death (PICD) in monocytes [130]. Similarly, MMP9-mediated processing of interleukin-2 receptor α (IL-2Rα or CD25), which plays an essential role for regulatory T cell (Treg) function, resulted in the abrogation of the efficacy of tumor-reactive cytotoxic lymphocytes by generating the soluble IL-2Rα/DC25 form that antagonized wild-type IL-2Rα [131]. MMP9 was also shown to be capable of releasing soluble fragments of the integrin β2 subunit (ITGB2, or CD18) from the monocyte/macrophage surface into the extracellular milieu. Truncated free ITGB2/CD18 forms were found to retain the ability to bind their ligands, such as intracellular adhesion molecule-1 (ICAM-1), fibrin, or collagen and thus function as receptor antagonists, limiting local inflammation [132,133].

Although the results of most studies indicate the protumor effect of MMP9, a study in which MMP9-deficient (MMP9^-/-^) mice were used surprisingly revealed that the reduction of MMP9 resulted in enhanced airway inflammation through an increase in the secretion of cytokines, such as IL-4 and IL-13, and chemokines, including eotaxin-1 (also known as CCL11) and macrophage-derived chemokine (MDC, or CCL22), as well as by heightened cell recruitment after allergen challenge [68]. A study on the effects of MMP9 loss in two independent mouse models of pancreatic neuroendocrine tumorigenesis (PNETs), RIP1-Tag2 (RT2), and pIns-MycER^TAM^RIP7-Bcl-x_L_ (Myc;BclXl) revealed increased invasiveness of MMP9-deficient tumors, which might confirm a positive role of MMP9 in regulating antitumor immunity [72]. In addition, it was observed that the treatment of immunodeficient mice bearing a human breast cancer with an adenoviral vector containing the MMP9 gene (AdMMP9) inhibited tumor growth and neoangiogenesis by inducing massive neutrophil infiltration into the cancer tissue, which resulted in an enhanced antitumor immune response [134].

### 3.3. MMP9 in Tumor Microenvironment Formation

Elevated levels of MMP9 are frequently observed in various types of cancers and are commonly considered to promote tumor growth and metastasis. Upregulated expression of MMP9 in tumor samples and serum of cancer patients was discovered to positively correlate with tumor stage and poor clinical outcome in a large variety of malignancies, including ovarian, colorectal, breast, and non-small cell lung cancer, suggesting that MMP9 could even be considered a potential prognostic marker for cancer patients [5,135,136,137,138]. Using the MMP9 transgenic mouse model, it was shown that overexpression of MMP9 in the liver significantly increased the susceptibility of transgenic animals to diethylnitrosamine (DEN)/phenobarbital (PB)-induced carcinogenesis [139]. There is a close relationship between increased MMP9 secretion and damage to tissue structures during cancer development. Due to its capacity to degrade extracellular protein components, MMP9 is involved in the reorganization of extracellular matrix proteins and the removal of physical barriers to cell migration. The disruption of the basement membrane (BM), which consists of a dense network of crosslinked laminins and collagens, accompanied by various glycoproteins and proteoglycans, facilitates not only the spread of invading cancer cells or the influx of immune cells into the tumor stroma but also the formation of new blood and lymphatic vessels that are necessary for supplying tumor tissue with nutrients and oxygen. Both angiogenesis and lymphangiogenesis are closely regulated by the angiogenic activators, including VEGF, bFGF, TGF-β, TNF-α, PDGF or IL-8, and EGFR/HER, which were shown to be related to MMP9 expression/activity, often associated with a positive feedback loop between MMP9 and most of these factors [140]. MMP9-dependent proteolytic processing of some of these factors was found to generate truncated functional isoforms that were able to induce endothelial cell motility [141]. Using the TIMER database to analyze the relationship between MMP9 and immune cell infiltration of cancers, Wang et al. found that MMP9 positively correlated with the infiltration of most of the immune cells, mainly Th1 cells, T follicular helper cells (Tfh), neutrophils, and macrophages, in a wide range of cancer types [138]. The physical changes in the ECM generated during neoplastic growth are also known to promote differentiation of normal fibroblasts to cancer-associated fibroblasts (CAFs). CAFs, which, unlike quiescent fibroblasts, exhibit enhanced metabolic activity, become the main creator of ECM [142]. By the production and secretion of large amounts of extracellular matrix proteins, CAFs play a key role in increasing tissue stiffness, which not only promotes the formation of more aggressive cancer phenotypes but also reduces the sensitivity to chemotherapy [142,143]. In addition, CAFs are known to secrete various growth factors, proinflammatory cytokines, and chemokines that promote angiogenesis and the recruitment of antitumor immune cells into the cancer stroma. The ability of MMP9 to modify the ECM also makes it an important factor when creating metastatic niches in secondary sites. Using the breast cancer model, blocking MMP9 with an active form-specific monoclonal antibody was found to abolish cancer cell colonization in the premetastatic lung niche [144]. It is known that components of the ECM serve as ligands for various cell receptors, and therefore, any change in the structure of the ECM may have a direct effect on the extracellular signaling system. The proteolytic activity of MMP9 was indicated as a key component of the regulation of cancer-related cell–cell and cell–ECM interactions by MMP9-dependent processing of E-cadherin and integrins and promoting EMT processes [75,102]. Using site-directed mutagenesis, Kim et al. identified transforming growth factor-β-induced protein (βig-h3) as another substrate of MMP9, and they discovered that MMP9-dependent cleavage of βig-h3 caused local release of this protein from the ECM and loss of its function, which consequently led to increased cell invasion [145]. 

The primary effects of MMP9 activity on cell migration, cancer-related invasion, and inflammation, as well as on tumor microenvironment formation, are summarized in Table 1.

### 3.4. Intracellular Activities of MMP9

More recent research has shown that MMP9 could process not only extracellular but also a wide range of intracellular proteins, including AMP-activated protein kinase *α* (AMPKα), connexin-43 (Cx43), heat shock proteins 60 and 70 (Hsp60 and Hsp70), poly (ADP-ribose) polymerase 1 (PARP1), and X-ray repair cross-complementing protein 1 (XRCC1) [146,147,148]. The intracellular activity of MMP9 was found to be localized to both the nucleus and cytoplasm as well as mitochondria. Reactive oxygen species (ROS) and reactive nitrogen intermediates, such as nitrogen dioxide (NO_2_) and peroxynitrite (ONOO_2_), produced during inflammation-associated tissue damage are thought to play a major role in the activation of intracellular MMPs, including MMP9, by oxidation-induced conformational changes that are able to activate MMPs even in their latent forms [149]. Intracellular MMP9 is considered to contribute to the pathogenesis of various diseases, but its role in tumorigenesis is unclear. Recently, nuclear MMP2 was shown to promote ribosomal RNA transcription and cell proliferation through cleavage of the N-terminal tail of histone H3 in osteosarcoma cell lines [150]. Earlier, Kim et al. found that MMP9-dependent proteolysis of the histone H3 N-terminal tail (H3NT) was involved in the regulation of genes that play critical roles in osteoclast differentiation [151]. It is possible that intracellular MMP9 may be linked to cancer growth, at least in part, by its participation in TLR-4-dependent regulation of innate immunity and inflammation [152].

### 3.5. Anticancer Effect of MMP9

Although MMP9 is generally known to enhance cancer progression, it is well established that MMP9 can also function as an inhibitor of cancer growth and metastasis. Enzymatic cutting of signaling molecules by MMP9 may lead to the generation of fragments that act as antagonists and block receptor activation. Using the mouse model of breast cancer, Bendrik et al. found that overexpression of MMP9 resulted in tumor regression by the generation of endostatin, a 20 kDa proteolytic fragment of type XVIII collagen that is known to inhibit angiogenesis [153]. Similarly, tumstatin, which is a 28 kDa matrikine released by MMP9 from the α3-chain of type IV collagen (COL4α3), was found to suppress cancer growth through αvβ3 integrin-mediated antiangiogenic activity. It was observed that MMP9-deficient mice were characterized by decreased levels of circulating tumstatin linked to an increased rate of tumor growth, and this effect was reversed upon restoration of the physiological concentration of tumstatin in blood [154]. Additionally, MMP9 was reported to have a tumor suppressor role in colitis-associated cancers (CACs) by the activation of Notch1-dependent intracellular signal transmitters that triggered cell apoptosis, cell-cycle arrest, and DNA damage [155,156].

## 4. MMP9 as an Anticancer Drug Target

### 4.1. Small-Molecule Inhibitors of MMP9

**Figure 3:**
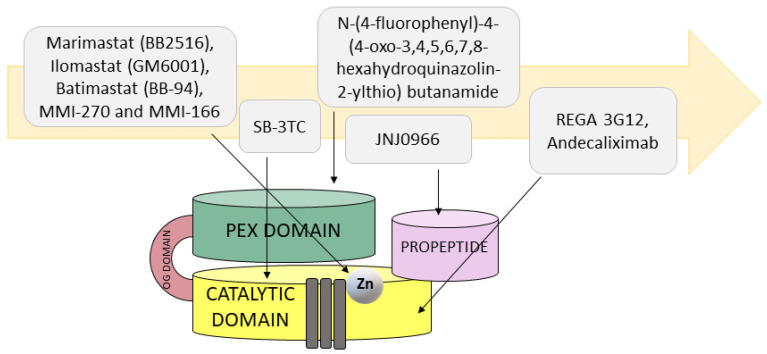
Alternative mechanisms for targeting MMP9 lead to enhanced specificity. Starting from the nonspecific small MMP9 inhibitors that chelate zinc ions (e.g., marimastat, ilomastat, and batimastat), through inhibitors targeting other than catalytic domain (N-(4-fluorophenyl)-4-(4-oxo-3,4,5,6,7,8-hexahydroquinazolin-2-ylthio) butanamide; JNJ0966) and ending with function blocking antibodies (REGA 3G12, andecaliximab), which in addition to blocking the activation of MMP9 also inhibit MMP9 activity and substrate binding. The yellow arrow symbolizes increasing specificity of MMP9 inhibitors.

Due to its role in many processes related to tumor progression, MMP9 seems to be an attractive target for anticancer therapies. Along with the discovery of MMPs three decades ago, research into the development of potential anti-MMP drugs began immediately (Figure 3). The initial therapeutics were small inhibitors binding to the catalytic domain of the enzyme, mimicking peptide sequences of the MMP’s endogenous ligands. Examples of such factors are marimastat (BB2516, (2S,3R)-N4-[(1S)-2,2-dimethyl-1-[(methylamino)carbonyl] propyl]-N1,2-dihydroxy-3-(2-methylpropyl)butanediamide)), ilomastat (GM6001, N-[(2R)-2-(hydroxamidocarbonylmethyl)-4-methylpentanoyl]-L-tryptophan methylamide, also known as galardin), and batimastat (BB-94, (2R,3S)-N^4^-hydroxy-N1-[(1*S*)-2-(methylamino)-2-oxo-1-(phenylmethyl)ethyl]-2-(2-methylpropyl)-3-[(2-thienylthio)methyl]butanediamide) [157]. These designed inhibitors utilize a hydroxamic acid moiety to chelate the catalytic zinc ion and consequently inactivate metalloproteases. Unfortunately, the hydroxamic acid moiety also binds other metals in addition to zinc; therefore, efforts have been made to differentiate the catalytic zinc chelating groups in order to improve the selectivity for this ion. In this way, a new generation of nonpeptidic compounds, including MMI-270 (N-hydroxy-2(R)-[(4-methoxysulfonyl)(3-picolyl)-amino]-3-methylbutaneamide hydrochloride monohydrate, also known as CGS-27023A) and MMI-166 (Nα-[4-(2- phenyl-2H-tetrazole-5-yl) phenyl sulfonyl]-D-tryptophan) utilizing sulfonylated amino acid hydroxamates, that selectively inhibit both MMP2 and MMP9, was developed. Although no antiproliferative effects were found in preclinical studies, these inhibitors significantly impaired angiogenesis and metastasis while reducing the tumor mass in animal models of breast, endometrial, and malignant glioma [158,159]. Unfortunately, these and many other small nonselective inhibitors have failed in clinical trials due to their poor solubility, low oral bioavailability, and severe side effects, including musculoskeletal syndrome (joint stiffness, pain, tendinitis, and inflammation) [160].

In order to avoid the constraints associated with the first-generation MMP inhibitors, such as metabolic inactivation and metal chelation of other metalloproteinases, other groups, including carboxylates, thiols, sulfhydryls, and phosphoric acid derivatives, were introduced in place of the hydroxamic acid, which reduced the potency of the drug but influenced MMP isoform selectivity [161]. Additionally, resolving the crystallographic structure of some MMPs paved the way for the new generation of non-zinc binding inhibitors with various backbone structures. In the catalytic domain, the Zn^2+^ ion is surrounded by substrate-binding sites designated as unprimed “left hand” S3, S2, and S1 and primed (often referred to as “right hand” site) S1′, S2′, and S3′. Of these pockets, S1 differs most among the various MMPs in terms of the amino acid composition and pocket depth. Molecular modeling and experimental studies showed that introductions of an arylsulfonyl group to hydrazide analogs of ilomastat (GM6001) made it possible to obtained the most potent and selective MMP9 inhibitor, with IC_50_ = 3 nM. The backbone of this inhibitor occupies the S2, S1′, and S2′ pockets and chelates the zinc atom group and forms two further H-bonds with the amino acids Pro423 and Tyr425 and two other H-bonds with Gly189 and Leu191 [162]. 

In developing selective drugs toward gelatinases, a mechanism-based strategy was applied. In the case of the thiirane-based inhibitor SB-3CT (2-[[(4-phenoxyphenyl)sulfonyl]methyl]thiirane), the competitive mechanism relies on the thiirane ring coordinating with the zinc ion within the active site and the diphenyl moiety binding to the S1′ deep subsite of gelatinases [163]. The antitumor activity of SB-3CT was tested in a mouse model of T-cell lymphoma, characterized by high MMP9 expression, where a significant reduction in tumor growth was observed. In addition, SB-3CT inhibited gelatinolytic activity in metastasis-bearing livers and increased the survival of mice [164]. In the severe combined immunodeficient (SCID) human model of prostate cancer (PC) bone metastasis, SB-3CT was found to reduce intraosseous tumor growth, which was correlated with a decrease in intratumoral vascularity and bone degradation [165]. Targeting angiogenesis within the bone microenvironment by the inhibition of gelatinase activity using the highly selective SB-3CT inhibitor may be a key aspect in the development of a promising therapy for patients experiencing metastases of PC to the bone. There are increasing studies reporting associations between the MMP family and cancer immunotherapy; for example, in the case of combined treatment of tumor mouse models of B16F10 melanoma and Lewis lung carcinoma with the MMP2/9 inhibitor SB-3CT and anti-programmed death receptor 1 (PD-1) or anti-cytotoxic T cell antigen 4 (CTLA-4), an enhanced antitumor immune response was observed [166].

The new approach for highly selective inhibitors of MMP9, preventing the conversion of an inactive zymogen form of MMP9 into mature, catalytically active enzyme, made it possible to distinguish a small molecule known as JNJ0966 (N-[2-[(2-methoxyphenyl)amino]-4′-methyl[4,5′-bithiazol]-2′-yl]acetamide). Structural studies of the complex of proMMP9 lacking the fibronectin II domains with JNJ0966 indicate that JNJ0966 binds within a region of the activation cleavage site (between residues 103 and 108), causing reorientation of adjacent amino acid residues 107–109 with no effect on the structure of the catalytic domain. Subsequent functional analysis confirmed the selectivity and specificity of this compound to proMMP9, as well as its impact on the reduction of invasion through stimulated ECM in HT1080 cells. The observed decrease in neurological disability in experimental autoimmune encephalomyelitis (EAE) mice treated with NJ0966, similar to those treated with dexamethasone (positive control), may indicate high pharmacological potential for use of NJ0966 in other disorders such as neurdegenerative diseases and cancer [167].

Several non-active site small MMP9 inhibitors that are more specific and selective have been described. Dufour et al., by using an in silico docking approach, selected several ligands that specifically bind to the PEX domain of human MMP9. The lead compound with the chemical name N-[4-(difluoromethoxy)phenyl]-2-[(4-oxo-6-propyl-1H-pyrimidin-2-yl)sulfanyl]-acetamide was shown to bind to the PEX domain of MMP9 with nanomolar affinity, but had no apparent effect on other MMPs. The binding of this compound to the PEX domain led to the abrogation of the homodimerization of MMP9 and, subsequently, prevented the downstream signaling pathway controlling cell migration in HT-1080 and MDA-MB-435 cells. In human breast cancer xenograft models, this pyrimidinone derivative significantly reduced primary tumor growth and lung metastasis. Toward the development of more potent PEX domain ligands for potential clinical application, an in silico library of analogs of the above compound was generated [168]. Out of 14 synthesized and tested ligands, one, N-(4-fluorophenyl)-4-(4-oxo-3,4,5,6,7,8-hexahydroquinazolin-2-ylthio) butanamide, showing high nanomolar affinity (K_D_ = 320 nM) toward the PEX domain of human MMP9, was selected. This inhibitor, by binding to the PEX domain of MMP9, prevents its dimerization and thus association with both α4β1 integrin and the CD44 receptor, leading to EGFR/HER dissociation. The consequence of this was not only abrogation of MAPK/ERK1/2 signaling pathways but also decreased phosphorylation of Src kinase, and its effector proteins such as FAK and paxillin in HT1080 both led to decreased migratory potential of human fibrosarcoma HT1080 cells. Additionally, treatment of HT1080 cells with this potent inhibitor significantly reduced angiogenic and invasive potential in a chorioallantoic membrane (CAM) assay [169]. Recently, Yosef et al. developed a multispecific inhibitor (C9-PEX), which targets both the catalytic domain and the PEX domain of MMP9. The strategy relied on the naturally occurring inhibitor TIMP2 since it binds more strongly to MMP9 than to other MMPs. The mechanism of action of C9-PEX, through which it antagonizes the interaction of MMP9 with CD44, leads to the suppression of ECM degradation and subsequently cancer cell migration and invasion [170]. Even though many such synthetic small compounds with increased selectivity have been developed [171,172], their clinical utility has not yet been determined, and there are currently no MMP inhibitors (MMPIs) in clinical trials for cancer.

### 4.2. Inhibitory Antibodies for MMP9

Recent research has focused on the development of functional blocking antibodies that selectively target MMPs. Monoclonal antibodies bind to a specific site outside of the catalytic domain of particular MMPs with high affinity, making them highly potent and selective drugs. Some of those antibodies are successfully undergoing phase I, II, and III clinical trials. These approaches resulted in the development of several anti-MMP9, highly selective monoclonal antibodies. One of the first was REGA 3G12, which binds to the N-terminal part of the catalytic domain but does not interact with the Zn^2+^ ion [173,174]. Two other anti-MMP9 monoclonal antibodies, B0041, which targets human MMP9, and B0046, which inhibits stroma-derived mouse MMP9, were found to be highly effective in a HCT 116 mouse colorectal cancer (CC) xenograft model. Treatment of CC mice with selective human and mouse antibody inhibitors of MMP9 caused significantly reduced tumor growth and incidence of metastases in comparison with the isotype controls. The selectivity and potency (K_D_ = 0.168 nM) of this allosteric inhibition led to clinical trials with the generated humanized version GS-5745 (andecaliximab) antibody showing promising therapeutic potential in ulcerative colitis and solid cancers. Moreover, andecaliximab was well tolerated and did not cause side effects, such as the musculoskeletal toxicity syndrome, as was the case with the broad-spectrum MMP inhibitors [175]. Structural studies of the MMP9/GS-5745 complex revealed that the antibody binds to MMP9 between the propeptide and the catalytic domain distal to the active site. Moreover, GS-5745 inhibits the activity of MMP9 through two mechanisms. One is by binding to the pro-form of MMP9, which prevents the activation of the secreted form of MMP9, and another is through noncompetitive inhibition of MMP9 activity [176]. A combined therapy of chemotherapeutics, such as mFOLFOX6, with andecaliximab in a phase Ib study with gastric and gastroesophageal junction adenocarcinoma showed a significantly decreased level of free MMP9 [177]. In a phase III trial, the efficacy and safety of GS-5745 combined with mFOLFOX6 were investigated [178].

Recently published data indicated that MMP9 by proteolytic inactivation of key T-cell chemoattractant CXC receptor 3 ligands, including CXCL9, CXCL10, and CXCL11, impaired the trafficking of tumor-infiltrating lymphocytes into the tumor microenvironment, an effective immune escape mechanism for many tumors. A combined antitumor treatment containing the aforementioned andecaliximab coupled with nivolumab/anti-PDL1 (an immune checkpoint inhibitor) in a syngeneic HER2-driven breast cancer (HC11-NeuT) tumor model drives intratumoral T-cell receptor diversity and promotes the antitumor response by increasing the protein level of Th1-type cytokines and infiltration of CD3+ T cells, including effector memory CD4 and CD8 positive T cells, into tumors [179]. The effectiveness of this combined therapy is currently being tested in phase II clinical trials in adult patients with unresectable or recurrent gastric or gastroesophageal junction adenocarcinomas (ClinicalTrials.gov Identifier: NCT02864381). Furthermore, the therapeutic efficacy of a highly specific anti-MPP9 antibody was observed in a combined treatment with the nab-paclitaxel plus gemcitabine (NPT + GEM) standard cytotoxic drugs in preclinical models of pancreatic cancer. Analysis of tumor tissue obtained from intraperitoneal xenografts after a combined treatment with an anti-MMP9 antibody and NPT revealed a decrease in the levels of stromal and EMT markers, such as smooth muscle alpha-actin (αSMA or ACTA2), collagen I, and vimentin in comparison with NPT + GEM therapy alone. Moreover, an anti-MMP9 antibody significantly reduced ascites and the tumor burden in the pancreas and improved the progression-free survival (PFS) in patients treated with NPT + GEM [180].

### 4.3. Natural Products with Anti-MMP9 Activity

A separate group of metalloproteinase inhibitors consists of naturally occurring compounds, which include alkaloids, flavonoids, and polyphenols derived from plants. One of them is silibinin A, a compound isolated from milk thistle seeds, which binds the active site of MMP9 and inhibits TPA (12-O-tetradecanoylphorbol-13-acetate)-induced MMP9 and COX-2 expression via inhibition of the Raf/MEK/ERK pathway in breast cancer cells [181]. Another example is gallic acid (GA, also known as 3,4,5-trihydroxybenzoic acid), a polyhydroxylphenolic compound present in various plants and fruits, showing anticancer effects on many types of solid tumors, including gastric, colon, and prostate cancers, as well as leukemia [182]. In human erythroleukemia K562 cells, GA reduces the expression of MMP2 and MMP9 through Bcr/Abl downregulation and JNK1 inactivation [183]. Many compounds from marine sources have also shown strong inhibition of MMPs. Methanolic extracts from the marine red alga *Corallina pilulifera,* for example, reduced UV-induced MMP2 and MMP9 expression in human dermal fibroblast cells. Similarly, fucoidan extracts from the seaweed *Cladosiphon novae-caledoniae* were shown to inhibit the invasion of human fibrosarcoma HT1080 cells via the suppression of MMP2 and MMP9 activity. Furthermore, fucoidan affected angiogenesis by the inhibition of VEGF expression and secretion in cervical cancer [184]. In order to search for new natural MMP9 inhibitors, the pharmacophore modeling and simulation approach was applied to screen the natural compound database. Revealed by these studies, hinokiflavone from *Juniperus communis* is characterized by stable interaction with an S1 loop of MMP9’s active site and inhibitory potential with IC_50_ = 43.08 µM in gelatinolytic inhibition assay and zymography. These results provide the basis for further research on hinokiflavone as a potential agent for cancer metastasis [185].

### 4.4. RNAi Therapeutics

Another approach toward a more specific targeting MMP9 activity relay on a highly precise mechanism of sequence-directed gene silencing or knockdown utilizes short 21–23 bp interfering RNA (siRNA), a double-stranded form of RNA that specifically binds its target in mRNA [186,187]. The mechanism of gene silencing through interfering RNAs involves either transient transfection of cells (short interfering RNAs (siRNAs)) or stable integration into the cell genome (short hairpin RNAs (shRNAs)).

RNAi-mediated silencing of MMP9 in B16 mouse melanoma cells significantly reduced migration and invasion, and furthermore, intratumoral injection of MMP9 siRNA into a C57BL/6 mouse tumor model resulted in the inhibition of tumor growth and reduced lung and liver metastasis, indicating MMP9 as a potential therapeutic target for malignant melanoma [188].

Silencing of siRNA genes for cathepsin B and MMP9 in glioblastoma SNB19 cells cocultured with microvascular endothelial cells manifested as inhibition of microvessel network formation induced by tumor cells. Furthermore, siRNA showed suppressive effects in the spheroid invasion assay. In vivo studies using nude mice confirmed the suppressive effect of RNAi for cathepsin B and MMP9 on malignant glioma tumor growth [189].

The therapeutic potential of siRNA-mediated targeting of MMP9, urokinase plasminogen activator receptor (uPAR), and cathepsin B, which play a major function in ECM degradation, was observed both in vivo and in vitro studies of prostate cancer. Downregulation of these proteases, inhibited the proliferation, invasion, in vitro angiogenesis, and wound-healing migration properties of PC3 and DU145 prostate cancer cell lines. Cells transfected with siRNA exhibited apoptotic features in TUNEL assay and fragmentation of cellular DNA in DNA laddering assay. Upregulation of proapoptotic proteins, such as Bax and caspases, and downregulation of antiapoptotic XIAP and Bcl-2 were revealed in Western blot analysis. The results were confirmed via in vivo experiments, where xenograft mice treated with siRNA were characterized by significant reduction in tumor size and volume in comparison with control mice and increased expression of FAS-L and cleaved caspase-8 in immunohistochemical analysis of prostate tumor sections [190].

An important issue in the use of RNAi as a potential anticancer drug is how to effectively introduce siRNA into specific cancer cells or tissues [191], nanoparticles [192], ultrasound-mediated microbubble destruction [193], or exosomes [194] have been developed, future studies are required on the biocompatibility, biodegradability, and safety of delivery systems in vivo. With the development of an appropriate delivery vehicle, the prospect of RNAi usage for MMP9 as cancer therapeutics in clinical trials seems probable. A summary of the selected MMP9 inhibitor drugs and their clinical outcome is presented in Table 2.

## 5. Conclusions and Future Perspectives

Recent studies have emphasized the role of MMP9 as a decisive contributor to the processes related to cancer tissue formation, growth, and malignancy. First of all, the proteolytic properties of MMP9 allow the degradation of structural proteins and remodeling of ECM that facilitate the passage of cancer cells through BM and, therefore, the invasion of adjacent healthy tissues. Additionally, extensive studies on substrate identification have revealed that MMP9 also participates in the release or activation of many different bioactive molecules, such as growth factors, chemokines, cytokines, and matrikines, that are responsible for cell migration, differentiation, and survival as well as immune response, angiogenesis, or tumor microenvironment formation. Increased levels of MMP9 have frequently been observed in various types of cancers and, in most cases, correlated positively with tumor stage and poor clinical outcome. This makes MMP9 an attractive target for cancer therapies. However, efforts to develop safe and effective drugs that selectively target MMP9 have been difficult due to the highly conserved active site among members of the MMP family and dose-limiting toxicity and side effects. Therefore, recent research led to the development of a functional blocking antibody that selectively deactivates MMP9, the specific antibody andecaliximab, currently in clinical trials in participants with advanced solid tumors. On the other hand, despite being considered to mediate protumorigenic effects, MMP9 was also found to enhance the regression of certain tumors.

Future research should focus more on a deeper understanding of mechanisms by which MMP9 contributes to the growth, progression, and spread of cancer that will promote the development of the next generation of therapeutics for MMP9-targeted therapy for cancer.

## Figures and Tables

**Table 1:** The effect of MMP9 on tumor progression.

Target	Effect of MMP9	Biological Consequences in Cancer	References
E-cadherin	Releasing the sE-cad fragment	Disruption of tight junction (TJ) integrity; cell dissociation; promotion of the EMT process; activation of EGFR, HER, and IGF-1R-dependent signaling pathways (MAPK, PI3K/Akt, and mTOR)	[75,83,84]
claudin-5	Degradation and loss of function	Disturbances of cellular polarity and epithelial barrier function, disruption of tight junction (TJ) integrity through the NF-κB signaling pathway	[93]
CD44	Formation of the CD44-MMP9 complex	Increasing the concentration and proteolytic activity of MMP9 against: (1) type IV of collagen to direct the migration of cancer cells, (2) TGF-β to promote cancer-associated fibroblast (CAF) differentiation and stimulate FN expression; initiating cross-talk between CD44 and HER-1 and triggering the activation of downstream effectors for cell migration; regulation of migratory potential and invasiveness of cancer cells	[94,95,97,98,100,101,102,103,104]
TLR-4, TrkA, EGFR/HER, and IR	Formation of a signaling platform	Induction of inflammation triggered by endogenous danger-associated molecular pattern (DAMP) molecules, increasing the tumorigenic potential of cancer cells, promoting immune evasion	[106,107,108,109,110,111,112]
IL-8/CXCL8	Releasing the truncated IL-8(7-77) form	Increasing the migration and activity of immune cells by activating the FAK/Akt/FOXO3A pathway, promoting the trafficking of neutrophils and MDSCs into the tumor stroma, inducing the EMT process, increasing the expression of MMP9 in cancer cells, increasing metastatic potential, promoting neutrophil degranulation, enhancement of angiogenic activity	[114,115,118,119]
CXCL5/ENA-78	Releasing truncated fragments	Activation of the EMT process by the ERK/GSK-3β/Snail pathway	[120]
CXCL11/I-TAC	Releasing the CXCL11/I-TAC(5-73) fragment	Inhibiting the antitumor immune response by acting as a natural antagonist of CXCR3	[122]
CXCL1/GRO-α, CXCL4/PF-4, CXCL7/CTAP-III, and CXCL12/SDF-1	Degradation and loss of function	Loss of chemotactic capacity and inhibition of the antitumor immune response	[120,121,122,123,126]
TNF-α and IL-1β	Releasing mature forms of TNF-α and IL-1β	Activation of signaling pathways that enhance cell proliferation and survival	[114,128,129]
TNFR1 and FAS/APO-1/TNFRSF6 death receptor	Cleavage of extracellular regions	Reduction of chronic inflammation by downregulation of cell-contact-related phagocytosis-induced cell death (PICD) in monocytes	[130]
IL-2Rα	Generating the soluble IL-2Rα/DC25 form	Abrogation of the efficacy of tumor-reactive cytotoxic lymphocytes antagonized with wild-type IL-2Rα	[131]
ITGB2	Releasing soluble fragments	Reducing local inflammation by maintaining the ability to bind ligands, such as ICAM-1, fibrin, or collagen, and acting as receptor antagonists	[80,81,82,132,133]
ECM proteins (i.e., laminins, collagens, and FN)	Degradation and releasing signaling fragments	Facilitating the spread of invading cancer cells and migration of immune cells, stimulation of angiogenic activators including VEGF and bFGF, promoting the differentiation of normal fibroblasts to CAFs, creating metastatic niches in secondary sites, promoting invasion by activating the FAK-Src-related signaling pathways due to the binding MMP9-degraded FN to αvβ6 and α5β1 integrins	[80,81,82,138,140,141,142,144]
βig-h3	Degradation and loss of function	Increasing the invasive potential of cancer cells	[145]

**Table 2:** Specificity and clinical research of selected MMP9 inhibitor drugs.

Group	Name/Description	Target	Clinical Outcome	References
Small inhibitors	Marimastat (BB2516, (2S,3R)-N4-[(1S)-2,2-dimethyl-1-[(methylamino)carbonyl] propyl]-N1,2-dihydroxy-3-(2-methylpropyl)butanediamide)	Catalytic domain (zinc chelator)	Cancelled in phase III clinical trials	[157]
	Ilomastat (GM6001, N-[(2R)-2-(hydroxamidocarbonylmethyl)-4-methylpentanoyl]-L-tryptophan methylamide, also known as galardin)	Catalytic domain (zinc chelator)	Cancelled in phase II clinical trials	[157]
	Batimastat (BB-94, (2*R*,3*S*)-*N*^4^-hydroxy-*N*1-[(1*S*)-2-(methylamino)-2-oxo-1-(phenylmethyl)ethyl]-2-(2-methylpropyl)-3-[(2-thienylthio)methyl]butanediamide)	Catalytic domain (zinc chelator)	Cancelled in phase III clinical trials	[157]
	MMI-270 (*N*-hydroxy-2(R)-[(4-methoxysulfonyl)(3-picolyl)-amino]-3-methylbutaneamide hydrochloride monohydrate, also known as CGS-27023A)	Catalytic domain (zinc chelator)	Cancelled in phase I clinical trials	[157,158]
	MMI-166 (Nα-[4-(2- phenyl-2H-tetrazole-5-yl) phenyl sulfonyl]-D-tryptophan)	Catalytic domain (zinc chelator)	Preclinical studies	[158,159]
	SB-3CT (2-[[(4-phenoxyphenyl)sulfonyl]methyl]thiirane)	Catalytic domain (zinc chelator)	Preclinical studies	[163]
	JNJ0966 (*N*-[2-[(2-methoxyphenyl)amino]-4′-methyl[4,5′-bithiazol]-2′-yl]acetamide)	Zymogen activation	Preclinical studies	[167]
	N-[4-(difluoromethoxy)phenyl]-2-[(4-oxo-6-propyl-1H-pyrimidin-2-yl)sulfanyl]-acetamide	PEX domain	Preclinical studies	[168]
	N-(4-fluorophenyl)-4-(4-oxo-3,4,5,6,7,8-hexahydroquinazolin-2-ylthio) butanamide	PEX domain	Preclinical studies	[168]
	C9-PEX	Catalytic domain and PEX domain	Preclinical studies	[170]
Inhibitory antibodies	REGA 3G12	N-terminal region of catalytic domain but not the Zn^2+-^binding site	Preclinical studies	[174]
	B0041	Zymogen activation and catalytic domain distal to active site	Preclinical studies	[175]
	B0046	Zymogen activation and catalytic domain distal to active site	Preclinical studies	[175]
	GS-5745 (andecaliximab)	Zymogen activation and catalytic domain distal to active site	Phase I, II, and III clinical trial solid tumors and phase III gastric adenocarcinoma (combined therapy of GS-5745 with mFOLFOX6) and phase II clinical trials gastric and gastroesophageal junction adenocarcinomas (GS-5745 coupled with nivolumab)	[176,177,178,179]
Naturally occurring inhibitors	Silibinin A, a compound isolated from milk thistle seeds	N/A	Preclinical studies	[181]
	Gallic acid (GA), also known as 3,4,5-trihydroxybenzoic acid	N/A	Preclinical studies	[182]
	Methanolic extracts from the marine red alga *Corallina pilulifera*	N/A	Preclinical studies	[184]
	Fucoidan extracts from the seaweed *Cladosiphon novae-caledoniae*	N/A	Preclinical studies	[184]
	Hinokiflavone from *Juniperus communis*	N/A	Preclinical studies	[185]
RNAi therapeutics		RNAi-mediated MMP9 gene silencing	Preclinical studies	[186,187,188,189,190]

## Supplementary Materials

The following supporting information can be downloaded at: https://www.mdpi.com/article/10.3390/cancers14071847/s1, Table S1: Members of the matrix metalloproteinase (MMP) family and their tissue inhibitors (TIMPs).

## Abbreviations

MMP—matrix metalloproteinase; ECM—extracellular matrix; PEX—hemopexin domain; OG—O-glycosylated domain; TIMP1/2—tissue inhibitor of metalloproteinases-1 and 2; AP-1—activator protein-1; NF-κB—nuclear factor-κB; Sp1—specificity protein 1; STAT3/5—signal transducer and activator of transcription 3 and 5; INF-α—interferon-α; IFNγ—interferon γ; TNF-α—tumor necrosis factor α; IL-1β—interleukin 1β; GM-CSF—granulocyte macrophage colony-stimulating factor; TGF-β—transforming growth factor β; PDGF—platelet-derived growth factor; bFGF—basic fibroblast growth factor; MAPK—mitogen-activated protein kinase; ERK1/2—extracellular signal-regulated kinase 1 and 2; JNK1/2—c-Jun N-terminal kinase 1 and 2; PI3K—phosphatidylinositol 3-kinase; mTOR—mammalian target of rapamycin; NGAL—neutrophil gelatinase-associated lipocalin; EMT—epithelial-to-mesenchymal transition; EGFR/HER—human epidermal growth factor receptor; IGF-1R—insulin-like growth factor-1 receptor; TIME—tumor immune microenvironment; IL-8/CXCL8—interleukin 8; AdMMP9—adenoviral vector containing the MMP9 gene; MMPI—MMP inhibitor.
