# Supplementary material for: MMP9: A Tough Target for Targeted Therapy for Cancer
Source: Cancers (Basel). 2022 Apr 6;14(7):1847. doi: 10.3390/cancers14071847 (PMC8998077; doi:10.3390/cancers14071847)
Supplement: Supplementary file 1 [file cancers-14-01847-s001.zip › cancers-1631193-supplementary.pdf]

cancers

MDPI

Table S1. Members of the matrix metalloproteinase (MMP) family and their tissue inhibitors (TIMPs).

PROTEIN	GENE	MW [Da]	Chromosomal localization	CATALYTIC ACTIVITY	Inhibitor
				COLLAGENASES	
**Interstitial collagenase, Collagenase 1, MMP1 (EC:3.4.24.7)**	MMP1	54007	11q22-q23	Collagen (I, II, III, VII, X), casein, entactin, laminin, pro-MMP1, -2, -9, alpha-macroglobulins and serpins	TIMP1, TIMP2, TIMP3, TIMP4
**Neutrophil collagenase, Collagenase 2, MMP8 (EC:3.4.24.34)**	MMP8 CLG1	53412	11q21-q22	fibrillar type I, II, and III collagens, interstitial collagens	TIMP1, TIMP2
**Collagenase 3, MMP13 (EC:3.4.24.-)**	MMP13	53820	11q22.3	fibrillar collagen (I, II, III, IV, X and XIV), fibronectin, TNC and ACAN, TGFB1, CCN2, gelatin, aggrecan, fibronectin, casein, laminin, perlecan, plasminogen activator 2, pro-MMP9, pro-MMP13, SDF-1, tenascin	TIMP1, TIMP2, TIMP3
				GELATINASES	
**72 kDa type IV collagenase, MMP2, Gelatinase A (EC:3.4.24.24)**	MMP2 CLG4A	73882	16q13	Gelatin, collagen (IV, V, VII, X), elastin, fibronectin, KISS, GSK3beta	histatin-5, TIMP1, TIMP2, TIMP3, TIMP4
**Matrix metalloproteinase-9, MMP9, Gelatinase B, GELB (EC:3.4.24.35)**	MMP9 CLG4B	78458	16q13	Gelatin, collagen (IV–VI, X), elastin, fibronectin, KiSS1, NINJ1	TIMP1, TIMP2, TIMP3, TIMP4
				STROMELYSINS	
**Stromelysin-1, MMP3, Transin-1 (EC:3.4.24.17)**	MMP3 STMY1	53977	11q23	fibronectin, laminin, gelatins of type I, III, IV, and V; collagens III, IV, X, and IX, and cartilage proteoglycans	TIMP1, TIMP2. TIMP4
**Stromelysin-2, Transin-2, MMP10 (EC:3.4.24.22)**	MMP10 STMY2	54150	11q22.3-q23	Collagens (I, III–V, IX and X), gelatin, casein, aggregan, elastin, MMP-1,8	TIMP1, TIMP2
**Stromelysin-3, ST3, STL3, MMP11 (EC:3.4.24.-)**	MMP11 STMY3	54589	22q11.2	Elastin, gelatin, collagen I, IV, fibronectin, laminin, vitronectin, proteoglycan, B chain of insulin	TIMP1, TIMP3
				MEMBRANE-TYPE MMPs	
**Matrix metalloproteinase-14, MMP14, Membrane Type 1-Matrix Metalloproteinase, MT1-MMP, MTMMP1 (EC:3.4.24.80)**	MMP14	65894	14q11q-12	Collagen, progelatinase A, PTK7, MMP15, ADGRB1, collagenase 3, aggrecan	TIMP2, TIMP3

**Matrix metalloproteinase-15, MMP15, Membrane Type 2-Matrix Metalloproteinase, MT2-MMP, MTMMP2 (EC:3.4.24.-)**	MMP15	75806	16q13	progelatinase A	TIMP2, TIMP3
**Matrix metalloproteinase-16, MMP16, Membrane Type 3-Matrix Metalloproteinase, MT3-MMP, MTMMP3 (EC:3.4.24.-)**	MMP16<br>C8orf57<br>MMPX2	69521	8q21	collagen type III and fibronectin, progelatinase A	TIMP1, TIMP2
**Matrix metalloproteinase-17, MMP17, Membrane Type 4-Matrix Metalloproteinase, MT4-MMP, MTMMP4 (EC:3.4.24.-)**	MMP17<br>MT4MMP	66652	12q24.3	pro-TNF-alpha, progelatinase A, fibrin, pro-TNF-alpha	
**Matrix metalloproteinase-24, MMP24, Membrane-type-5 matrix metalloproteinase, MT5-MMP, MTMMP5 (EC:3.4.24.-)**	MMP24<br>MT5MMP	62553	20q11.2	N-cadherin, fibronectin, dermatan sulfate and chondroitin sulfate proteoglycans, progelatinase A	
**Matrix metalloproteinase-25, MMP25, Membrane-type-6 matrix metalloproteinase, MT6-MMP, MTMMP6 (EC:3.4.24.-)**	MMP25<br>MMP20<br>MMPL1<br>MT6MMP	58938	16p13.3	progelatinase A	
**MATRILYSINS**					
**Matrilysin, Matrin, Matrix metallo-proteinase-7, MMP7, Pump-1 pro-tease, Uterine metalloproteinase (EC:3.4.24.23)**	MMP7<br>MPSL1<br>PUMP1	24144	11q21-q22	casein, gelatins of types I, III, IV, and V, and fibronectin. Procolla-genase, insulin	TIMP1, TIMP2, TIMP3, TIPM4
**Matrix metalloproteinase-26, MMP26, Endometase, Matrilysin-2 (EC:3.4.24.-)**	MMP26	29708	11p15	collagen type IV, fibronectin, fibrinogen, beta-casein, type I gela-tin and alpha-1 proteinase inhibitor, progelatinase B	
**METALLOELASTASES**					
**Macrophage metalloelastase, macrophage elastase, metallopro-teinase-12, MMP12 (EC:3.4.24.65)**	MMP12<br>HME	54002	11q22.2-q22.3	Gelatin, collagen IV, pro-MMP9, elastin, insulin	TIMP1
**ENAMELYSIN**					

**Matrix metalloproteinase-20, MMP20, Enamel metalloproteinase, Enamelysin, (EC:3.4.24.-)**	MMP20	62553	11q22.3	aggrecan and the cartilage oligomeric matrix protein (COMP)	
			**Other MMPs**		
**Matrix metalloproteinase-19, MMP19, Matrix metalloproteinase RASI, Matrix metalloproteinase-18, MMP18 (EC:3.4.24.-)**	MMP19<br>MMP18<br>RASI	57356	12q14	collagen type IV, laminin, nidogen, nascin-C isoform, fibronectin, and type I gelatin, aggrecan and cartilage oligomeric matrix protein	TIMP2
**Matrix metalloproteinase-21, MMP21 (EC:3.4.24.-)**	MMP21	65042	10q26.3	alpha-1-antitrypsin	
**Matrix Metalloproteinase 23A, Matrix Metalloproteinase 22, MMP22, Femalysin, MIFR-1, Matrix metalloproteinase-21, MMP21 (Pseudogene)**	MMP21/P<br>MMP22<br>MMP23A<br>MMP23B				TIMP2
**Matrix metalloproteinase-27, MMP27 (EC:3.4.24.-)**	MMP27<br>UNQ2503/PRO5992	59025	11q24	fibronectin, laminin, gelatins and/or collagens	
**Matrix metalloproteinase-28, MMP28, Epilysin (EC:3.4.24.-)**	MMP28<br>MMP25<br>UNQ1893/PRO4339	58938	17q21.1	casein	
